# High-speed reservoir computing using photonic integrated circuit optical parametric oscillators

**DOI:** 10.1126/sciadv.aeb3077

**Published:** 2026-09-18

**Authors:** Midya Parto, Gordon H. Y. Li, Ryoto Sekine, Robert M. Gray, Luis L. Ledezma, James Williams, Arkadev Roy, Alireza Marandi

**Affiliations:** ^1^ Department of Electrical Engineering, California Institute of Technology, Pasadena, CA 91125, USA.; ^2^ Physics and Informatics Laboratories, NTT Research Inc., Sunnyvale, CA 94085, USA.; ^3^ CREOL, The College of Optics and Photonics, University of Central Florida, Orlando, FL 32816, USA.; ^4^ Department of Applied Physics, California Institute of Technology, Pasadena, CA 91125, USA.

## Abstract

Over the past decade, deep learning has led to disruptive advancements with key applications in computer vision, natural language processing, and predictive analytics. With the increasing prevalence and adoption of deep learning algorithms, the quest for hardware solutions that can efficiently process data in real time with high speeds and low latencies has come to the forefront of research. On-chip photonic neural networks offer a promising platform that leverage high bandwidths and low propagation losses associated with light to perform analog deep learning computations. However, nanophotonic circuits are yet to achieve the required linear and nonlinear operations simultaneously in an all-optical and ultrafast fashion. Here, we report a high-speed photonic integrated circuit (PIC)–based reservoir computer using an optical parametric oscillator (OPO) fabricated on thin-film lithium niobate. We apply the PIC-based OPO computing system for a variety of benchmark tasks including chaotic time series prediction, nonlinear error correction in a noisy communication channel, and noisy waveform classification, achieving >93% accuracies at an operating clock rate of ∼10 gigahertz in all cases. Our OPO network can allow for subnanosecond latencies when implemented in an end-to-end all-optical fashion, a timescale that is shorter than a single clock cycle in state-of-the-art digital electronic processors. By circumventing the need for optical-electronic-optical conversions, our high-speed PIC-based reservoir system paves the way for the next generation of compact, energy-efficient, all-optical neural networks with ultralow latencies.

## INTRODUCTION

Deep neural networks (DNNs) (*1*) have revolutionized modern data processing with transformative results across numerous fields ranging from fundamental sciences to automotive and health care industries to generative art (*2*–*4*). As deep learning algorithms become more prominent, traditional computing hardware proves to be increasingly less suitable for their implementation. This incompatibility poses a formidable challenge for the future of artificial intelligence (AI) and originates from several characteristics of current computing architectures. The substantial growth of the compute required by recent deep learning models far surpasses the current trend in the advancement of digital electronic processors (*5*). The resulting gap becomes even more acute in many practical scenarios that require real-time processing where ultralow latencies are critical. Providing viable solutions to these challenges has brought the quest for efficient and high-speed computing hardware to the forefront of research across various disciplines to drive disruptive AI technologies.

Against this backdrop, photonic neural networks (PNNs) have recently emerged as promising candidates that can harness high bandwidths offered by optical systems to provide ultrafast operation while maintaining high energy efficiencies facilitated by the low-loss propagation of light (*6*–*10*). In particular, recent advances in nanofabrication and photonic integrated circuits (PICs) have provided a fertile ground for implementing light-based AI architectures on compact chip-scale devices (*6*, *7*, *11*–*16*). DNNs consist of many layers of linear operations such as matrix multiplications or convolutions interleaved by nonlinear activation functions. Existing integrated PNNs use various architectures such as Mach-Zehnder inteferometers (*17*), as well as microring resonator arrays to accelerate the linear operations necessary for deep learning (*11*, *13*, *18*). Typically, optoelectronic components provide the nonlinear activation functions in PNNs. Alternatively, one can realize the nonlinear operations using all-optical nonlinearities (*19*) such as phase change materials (*20*–*22*) or optical parametric processes (*23*, *24*). This latter approach can therefore eliminate the need for successive optical-electronic-optical (OEO) conversions and provide a route for true all-optical integrated neural networks with ultrashort latencies beyond those attainable by hybrid optoelectronic architectures. However, despite notable progress in PNNs (*25*), achieving a unifying platform that simultaneously provides both linear multiply-accumulate operations and ultrafast nonlinear activation functions in nanophotonic circuits has remained elusive.

**Fig. 1.**
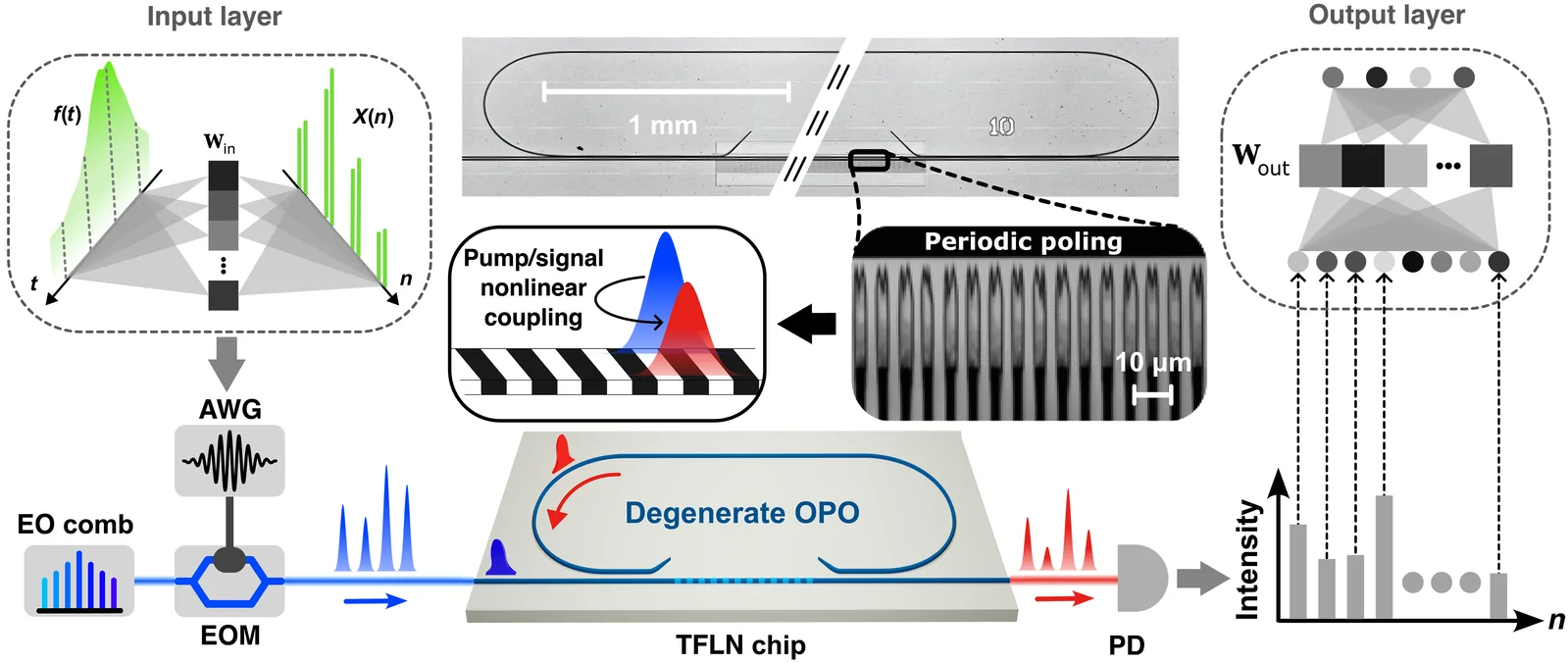
Nanophotonic OPO-based reservoir computer. Schematic view of the nanophotonic OPO used in our experiments for reservoir computing. The top panel depicts the microscope image of the device [more information about the chip design can be found in (*40*)]. The central part of the racetrack resonator is removed for clarity. The structure is designed in such a way that only the degenerate signal/idler resonate inside the cavity. At the input, the optical pump generated from an EO comb at $f_{\text{rep}}\approx 10\;\text{GHz}$ and a center wavelength of ∼1045 nm is modulated by an EOM. The modulating signal is generated by an AWG that applies discrete data *X*(*n*) at ∼10 GSa/s. These data represent a time series that is obtained by sampling an arbitrary function *f*(*t*) in time, followed by masking with a randomized set of input weights **W**_in_, thus forming the input layer of our OPO neural network. The inset depicts a two-photon microscope image of the periodically poled region in the OPO cavity necessary for quasi–phase matching (QPM). Within this poled region, the generated signal pulses from previous roundtrips nonlinearly couple to the incoming pump pulses, collectively acting as a nonlinear recurrent network that connects the input layer to the output (the inset schematically displays this nonlinear coupling). At the output, the signal pulses at ∼2090 nm are separated from the pump using a fiber-based WDM filter and sent to a fast photodetector. The output layer of our OPONN is then set up by forming a weighted average of these detected pulses.

In this work, we address this challenge and demonstrate an integrated PNN that harnesses coherent optical pulse propagation in conjunction with ultrafast parametric nonlinear processes (*26*, *27*) to achieve ultralow-latency operations. To this end, we use an integrated optical parametric oscillator (OPO) fabricated on a thin-film lithium niobate (TFLN) chip to implement a reservoir computing neural network, which we dub OPONN. In our scheme, as shown in Fig. 1, masked sequential data are used to modulate the optical pulses of an electro-optic (EO) frequency comb used to synchronously pump the OPO. In response, the integrated OPO, which operates at degeneracy (*28*), generates signal pulses at the half-harmonic of the input pump. The optical feedback provided by the cavity on one hand and the parametric amplification on the other hand act upon the generated signal fields creating dynamics that occur on fast timescales and are akin to those associated with neurons in a recurrent neural network. We use our OPONN for machine learning on three benchmark tasks: (i) high-speed chaotic time series prediction involving the archetypal Lorenz system and Mackey-Glass system (MGS) of equations, (ii) compensating nonlinear distortions in a noisy communication channel, and (iii) waveform classification on noisy signals that are randomly chosen from three different classes of sinusoidal, rectangular, and sawtooth functions. In all cases, the OPONN achieved accuracies exceeding 93% while operating at ∼10-GHz clock rates. This clock rate is only limited by the electronic sources responsible for generating the input pump pulses and is not an inherent limit of our OPO-based neural network, which is operable with femtosecond pulses (*29*). By realizing the linear operations and the nonlinear activation functions in the optical domain, our implementation also eliminates the need for successive OEO conversions that are typically required in state-of-the-art PNN architectures.

## RESULTS

Figure 1 illustrates the OPO-based neurmorphic reservoir computer. At the heart of the system is an on-chip OPO, which is fabricated on an x-cut TFLN wafer with a silica buffer layer. It consists of a dispersion-engineered periodically poled lithium niobate (PPLN) section that provides broadband quasi–phase matching (QPM) between the pump at 1045 nm and the generated signal and idler waves. The cavity is formed by two input/output adiabatic couplers, which are designed to only allow the signal/idler modes to resonate within the cavity (*28*). Throughout our experiments, the OPO is operated in the degenerate regime. We synchronously pump the OPO using transform-limited pulses with a duration of ∼2 ps generated by an EO comb with a repetition rate of ∼10 GHz matching the cavity free spectral range. The input layer of the neural network is formed using an EO modulator (EOM) driven by an arbitrary waveform generator (AWG) operating at ∼10 gigasamples per second to prepare the masked sequential data fed into the OPO as the pump pulses. During successive cycles defined by the roundtrip time of the OPO cavity, the input pump pulse and the signal pulse traveling in the cavity overlap in the PPLN region. The signal pulse carries information from the previous signals due to the feedback provided by the cavity. In this respect, the resonator serves as an optical memory. Followed by this coherent interference between the signal and pump pulses, the PPLN element acts as a nonlinear neuron that generates a signal pulse, which is a nonlinear function of its inputs, i.e., the current pump pulse and the previous signal pulse. In this respect, the OPO provides an all-optical implementation of a recurrent reservoir. Last, the weighted average of the signal pulse intensities resolved by a fast detector forms the output layer of the OPONN (Fig. 1).

### Low-latency time-domain signal processing using OPONN

**Fig. 2.**
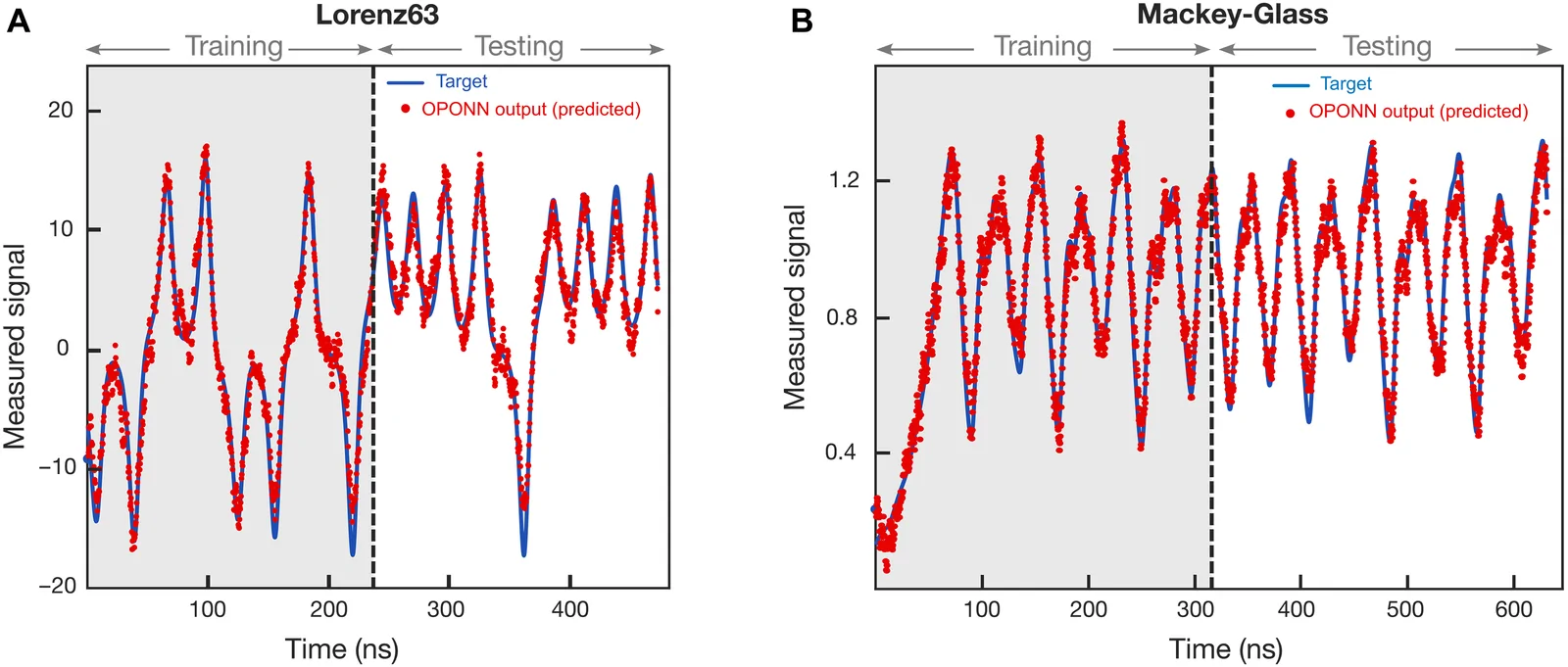
Chaotic time series prediction using OPONN. (**A**) Numerically calculated values of the *x*(*t*) signal associated with the Lorenz63 model (target) and the experimentally measured signal at the output of our OPONN (predicted). As shown in the figure, the entire span of the signal with a ∼500-ns duration is split into two equal sections for training (the shaded area) and testing. As evident, the predictions of the OPONN closely match the target values. (**B**) Similar results for the MGS of equations in the chaotic regime.

We used the OPONN to perform machine learning on a variety of benchmark tasks that involve time-domain signals. The first task is to predict the evolution of a chaotic time series generated by the Lorenz63 model proposed by Lorenz in 1963 (*30*) to describe atmospheric convection. This is governed by a three-dimensional system of coupled nonlinear differential equations $\dot{x}=10\left(y-x\right),\dot{y}=x\left(28-z\right)-y,\dot{z}=\mathrm{xy}-8z/3$, where *x*, *y*, and *z* denote physical observables associated with the convective current and the temperature variations in different spatial directions. For the set of parameters and the initial conditions chosen here, the Lorenz system exhibits deterministic chaos that is manifested by an aperiodic trajectory in the phase space known as a strange attractor. Under these conditions, the evolution of the observables show a marked sensitivity to the initial conditions, hence defying conventional methods of prediction. Our goal here is to train the OPONN to forecast the next time step of the signal *x*(*t*) based on its past history. To achieve this, we first sample the input data at a sampling rate of ∼10 GHz to obtain the discrete signal *u*(*n*). We then configure the network with $N_{\text{in}}=3$ and $N_{\text{out}}=10$ nodes in the input and the output layers, respectively. The input weights defined by the vector **W**_in_ are chosen randomly. We split the entire span of the available data into two equal periods of training and testing. Training is performed in silico by singular value decomposition to obtain the optimal output weight matrix **W**_out_ that results in the least estimation error during the training phase. In contrast to other types of DNNs that typically require computationally intensive training techniques such as backpropagation, our neural network can be trained considerably simpler with minimal computation requirements. Figure 2A shows measurement results obtained from experiments, where both the target and the predicted signals are displayed. To quantify the performance of the OPONN for this forecasting task, we calculate the normalized mean square error between the target and the predicted time series given as 0.07 ± 0.017 for this task.

As a second example, we consider another benchmark time series prediction task associated with the MGS, which was originally developed in the study of physiological control mechanisms that are known to exhibit a host of complex behaviors (*31*). The model is described by a class of nonlinear delay differential equations exhibiting dynamics that critically depend on the system parameters. Of particular interest is the scenario when the solutions become chaotic, signaling an abnormal physiological behavior associated with pathological cases. Here, we use our OPO network to forecast the future instances of the resulting chaotic time series. Figure 2B depicts the target MGS solution together with predicted values obtained by measuring the OPONN output, indicating a prediction error of NMSE=0.06±0.017.

### Nonlinear channel equalization

**Fig. 3.**
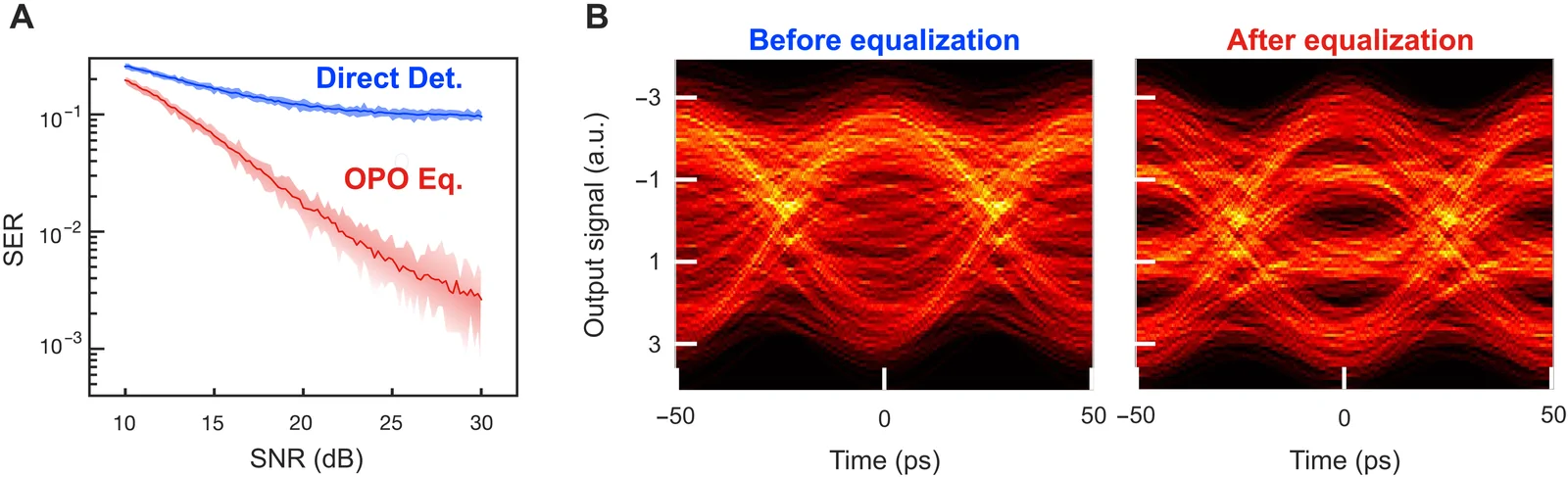
Nonlinear channel equalization of PAM4 signals using ultrafast nonlinear response of the OPO network. (**A**) Simulated SERs obtained using direct detection (Det.) versus those achieved after OPONN equalization (Eq.) for various SNR levels. (**B**) Experimentally obtained eye diagrams without (left) and with (right) OPONN equalization.

To demonstrate the versatility of our high-speed PIC reservoir computer, we next exploit it to compensate for nonlinear distortions that typically occur in a wireless communication channel. We assume that the input message to be transmitted contains a random stream of symbols *M*(*n*) of the four-level pulse-amplitude modulation (PAM4) format. This modulation scheme is of practical interest in data centers and is implemented for instance in state-of-the-art 800G transceivers. Upon entering the communication channel, the message is subjected to multiple adverse effects that degrade the fidelity of the received signal *S*(*n*) at the end of the channel. First, the presence of scatterers located around the path between the source and the receiver result in multipath fading that causes intersymbol interference among adjacent symbols. In addition, to overcome the losses that are incurred through the signal propagation, it is desirable to maximize the power of the transmitted signal by increasing the gain from the power amplifier in the output stage of the transmitter. This increase in the power is accompanied by nonlinear effects that distort the message. In addition, the signal-to-noise ratio (SNR) is diminished by various noise sources, including thermal fluctuations and the noise introduced by amplifiers. Previous studies have shown the potential of recurrent neural networks in correcting for these cumulative distortions (*32*–*34*). Our objective is to perform this error correction task by exploiting the OPONN. Here, $N_{\text{in}}=3$ and $N_{\text{out}}=21$ nodes are used in the input and output layers, respectively. To assess the success of our approach, we calculate the symbol error rate (SER) defined as the percentage of the symbols detected incorrectly at the receiver end. Simulation results predict that the OPONN can effectively correct these errors and significantly improve the SER (Fig. 3A). Our experiments corroborate this and show that a direct detection of the corrupted signal at the channel output results in a high SER of 19 ± 1.6% (Fig. 3B). In contrast, once trained, the OPONN is able to improve this result to SER=7±2.3%. To confirm the role of the nonlinear processing performed by the OPO in error compensation, we also considered a purely linear equalization applied directly to the input pump, for which a SER=11±1.8% was measured. Given the fact that the measured SNR of the OPO output was lower than that associated with the input pump (see the Supplementary Materials), this latter comparison clearly indicates the efficacy of the OPO network to harness ultrafast photonic nonlinear processes to perform this equalization task.

### Noisy waveform classification

**Fig. 4.**
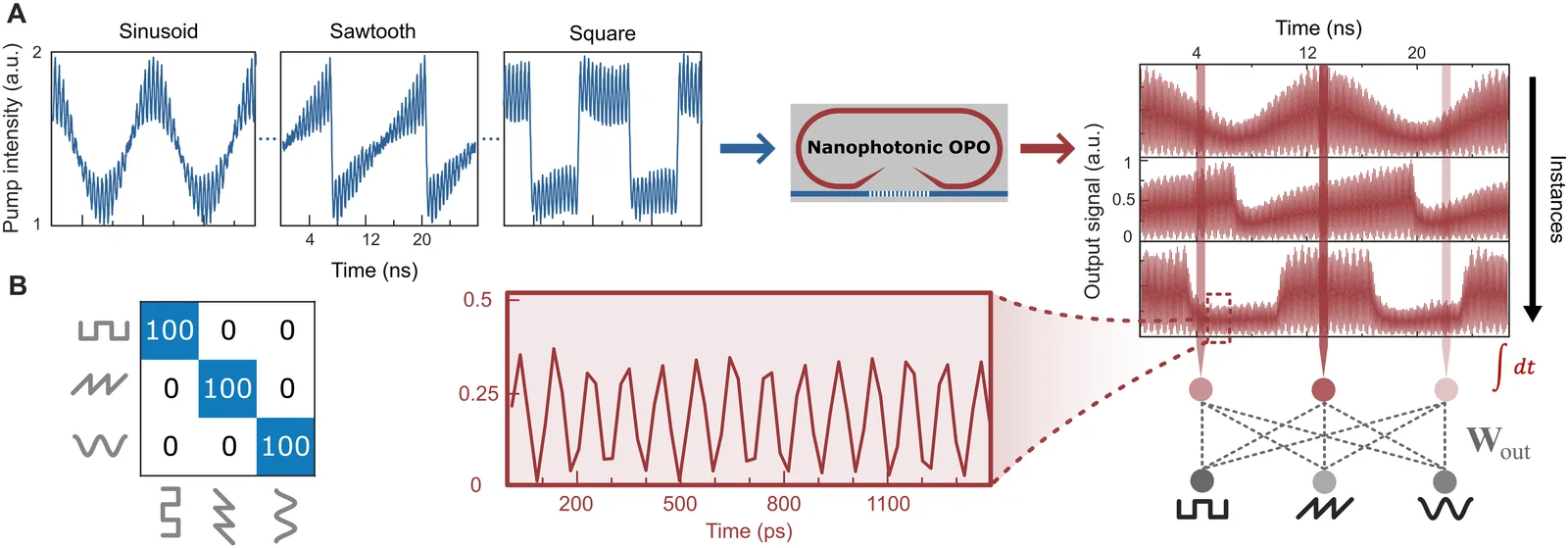
Noisy waveform classification task. (**A**) We use the AWG to encode samples representing 2 cycles of periodic waveforms randomly chosen from three different classes of sawtooth, sinusoid, and square signals with additive noise on the input pump (left). The measured signals at the output of the OPO associated with these waveforms are shown on the right. The output signals are sampled at three equally distant instances corresponding to three different nodes. The output layer of the OPO network is then set up by forming a fully connected layer between these nodes and the output nodes that are activated based on a winner-takes-all approach. (**B**) Using this simple architecture, we achieved 100% accuracy for this classification task.

One of the key applications in time-domain signal processing involves the classification of various waveforms, with numerous applications ranging from bioinformatics (*35*, *36*) to seismology (*37*). To showcase the generality of our approach, we next used the OPO neural network to distinguish among three classes of different waveforms randomly chosen from sinusoidal, square, and sawtooth signals that are contaminated with noise (Fig. 4A). Each waveform contains *N* = 50 samples constituting two consecutive cycles. For this experiment, the OPONN is set up with $N_{\text{in}}=5$ nodes at the input. At the output of the chip, the subharmonic signal generated by the OPO is sampled at three equidistant points, forming *K* = 3 output neurons (Fig. 4B). The output layer of the neural network consists of a fully connected layer with $N_{\text{out}}=3$ nodes. During the training period, the linear weights associated with this output layer **W**_out_ are optimized according to a winner-takes-all approach. We use a training set of 300 waveforms (100 from every class) and a different set of equal size for testing. In all cases, the OPONN was able to successfully classify all waveforms with 100% accuracy, despite the presence of significant noise levels at the input (Fig. 4B).

## DISCUSSION

In conclusion, we have demonstrated a high-speed photonic reservoir computer based on a PIC-based OPO operating at 10-GHz clock rates. Our approach leverages coherent optical pulse propagation in conjunction with ultrafast parametric nonlinear processes available in TFLN to perform the linear and nonlinear operations required in reservoir computing in the optical domain. Operating at ∼10-GHz clock rates, our OPO neural network can operate at subnanosecond latencies when implemented in an end-to-end all-optical fashion (see section S2), a timescale comparable to a single clock cycle in state-of-the-art digital electronic processors. Moreover, the ∼10-GHz clock rate implemented in our study is only limited by the electronic components of our current architecture. Since our integrated OPO is operable with femtosecond pulses (*29*), one can envision orders of magnitude higher clock rates using temporal interleaving of ultrashort pulses (*38*) in an all-optical implementation of our scheme. We showcased the power of OPONN in performing a variety of machine learning tasks that involve time series signals including chaotic time series prediction, nonlinear channel equalization, and noisy waveform classification. In all cases, the OPONN achieved success rates exceeding 93%. In particular, the 100% accuracy of our network for the waveform classification task encourages us to explore more difficult classification tasks such as those involving higher-dimensional classes in our future studies. By eliminating successive slow OEO conversions, our OPONN represents an important leap toward realizing integrated all-optical neural networks operating at unprecedented high speeds. Although our scheme represents a single computing node that processes serial data at the input, it can potentially be generalized to process many parallel channels of data using various multiplexing schemes such as time multiplexing (*38*) or coupled resonator (*39*) (see section S8). Moreover, the footprint of our OPO reservoir architecture can be significantly reduced via using integrated EOMs and EO comb generators (*40*) realized on TFLN.

## MATERIALS AND METHODS

Figure 1 shows a schematic of our experimental setup. We use an EO comb to generate optical pulses at 10-GHz repetition rate and center wavelength of 1045 nm with a pulse width of ∼2 ps to synchronously pump the integrated OPO. These pulses are passed through a booster optical amplifier, wave shaper, and ytterbium-doped fiber amplifier (YDFA) for amplification and dispersion compensation. The sequential data at the input layer are prepared by modulating the pump pulses using an intensity modulator driven by an AWG operating at 10 GSa/s. The modulated pump pulses are further amplified using a second YDFA before being coupled into the chip. Lensed fibers are used to couple the pump into the chip and to collect the emitted signal at the output. Our on-chip OPO is fabricated on an x-cut, 700-nm-thick TFLN wafer with a silica buffer layer. It comprises two input/output adiabatic couplers before and after the periodically poled section, which are designed to only allow the degenerate signal/idler modes to resonate within the cavity. The emitted signal pulses at 2090 nm are resolved using a fast biased detector (with 12-GHz bandwidth) and form the output layer of the OPONN. As evident from experimental measurements presented in fig. S2, the SNR of the detected pump pulses was significantly higher than that associated with the signal pulses.
