# Supplementary material for: High-speed reservoir computing using photonic integrated circuit optical parametric oscillators
Source: Sci Adv. 2026 Sep 18;12(38):eaeb3077. doi: 10.1126/sciadv.aeb3077 (PMC13588183; doi:10.1126/sciadv.aeb3077)
Supplement: Supplementary file 1 [file sciadv.aeb3077_sm.pdf]

Science Advances
AAAS

Supplementary Materials for

High-speed reservoir computing using photonic integrated circuit optical parametric oscillators

Midya Parto *et al.*

Corresponding author: Midya Parto, midya.parto@ucf.edu; Alireza Marandi, marandi@caltech.edu

Sci. Adv. **12**, eaeb3077 (2026)
DOI: 10.1126/sciadv.aeb3077

This PDF file includes:

Supplementary Text
Figs. S1 to S9
Tables S1 and S2

Supplementary Text

1 - Comparison to other physical recurrent neural networks

The concept of the OPONN is closely related to computing using physical recurrent neural networks (*42*), which exploits complex nonlinear dynamics in analog physical systems. In this section, we compare our OPONN with other experimentally demonstrated physical recurrent networks in different platforms based on various physical mechanisms including optoelectronic, magnetic, electrical, mechanical, chemical, atomic, and biological. The unique advantage of the OPONN is its exceptionally high computational clock rate, whereas other physical recurrent networks usually prioritize low power consumption. Here, we restrict our attention to experimentally demonstrated results. Although the types of computational operations and network architectures can vary greatly between physical recurrent networks, we consider the maximum effective computational clock rate (even if the implementation does not have a literal hardware clock as part of the design) to be given by the fastest reported input update rate or minimum time between successive operations in the physical recurrent layer. The maximum clock rate therefore relates to the theoretical minimum latency or fastest input signal time scales that the physical recurrent networks was demonstrated to respond to in real-time. The rough order-of-magnitude ranges for different categories of experimentally demonstrated physical recurrent networks and a corresponding state-of-the-art example for each category are shown in Fig. S3. A comparison of the representative state-of-the-art examples for each category of physical mechanisms of the physical recurrent networks is shown in Table .

To obtain a fair comparison, we can compare our results to those reported in Ref. (*43*) of the manuscript which features an effective clock cycle ($\approx 5GHz$) comparable to our demonstration. In that work, an accuracy of $\approx 90\%$ is reported in predicting the Santa Fe time series with the number of input nodes equal to $N_{in} = 1$. To improve this result, the authors then consider using a larger number of $N_{in} = 5$ to improve the accuracy to $\approx 94.5\%$. In comparison, we obtained accuracies of $\approx 93\%$ and $\approx 94\%$ in the Lorenz63 and the Mackey-Glass time series prediction tasks, respectively, by using $N_{in} = 3$ for the number of input nodes.

2 - All-optical output layer

In the main text experiments, we focused on demonstrating the ultrafast nonlinear recurrent layer of the OPONN, which comprises the bulk of the computation and primary bottleneck of neuromorphic processors. We chose to perform the linear output layer during post-processing on a conventional digital electronic computer for simplicity since many previous works have shown how to accelerate linear operations using optics. In this section, we propose a simple implementation for an end-to-end all-optical OPONN that does not rely on electronics for executing the linear output layer.

In the context of physical recurrent networks, the recurrent layer provides highly nonlinear and complex dynamics, and the linear output layer contains fixed weights trained by linear regression. In the OPONN, the linear output layer can be implemented in the same thin-film lithium niobate integrated platform as the recurrent layer. For example, Fig. S4 shows a multi-arm Mach-Zehnder interferometer that can perform a linear dot product for a single-channel output (e.g., for time series forecasting tasks) directly in the optical domain. The relative delays in each arm encode for the corresponding time-delayed recurrent variables, which are represented by the coherent amplitudes of ultrashort laser pulses. The electro-optical amplitude modulators in each arm encode the fixed weights of the linear output layer. Therefore, the recurrent variables can be measured during the training phase, then the optimal output layer weights can be determined using linear regression (usually on a digital computer), and finally used to set the bias voltages for the electro-optic amplitude modulators in the output layer. Once the output layer weights are determined, the computation can be performed entirely in the optical domain during testing or inference stages.

It should be emphasized that the role of the EOMs in the all-opitcal architecture of Fig. S4 is simply to provide the output weights that are determined in the training phase. These output weights are static, hence do not need to be dynamically updated. Because of this, such an implementation does not slow down the linear operation in the output layer. We note that as long as the corresponding linear weights are stored in an electronic memory, the presence of such EOMs is mandatory for the retrieval of information from the electronic memory to the optical domain. The only way to entirely eliminate the EOMs is to utilize all-optical memories.

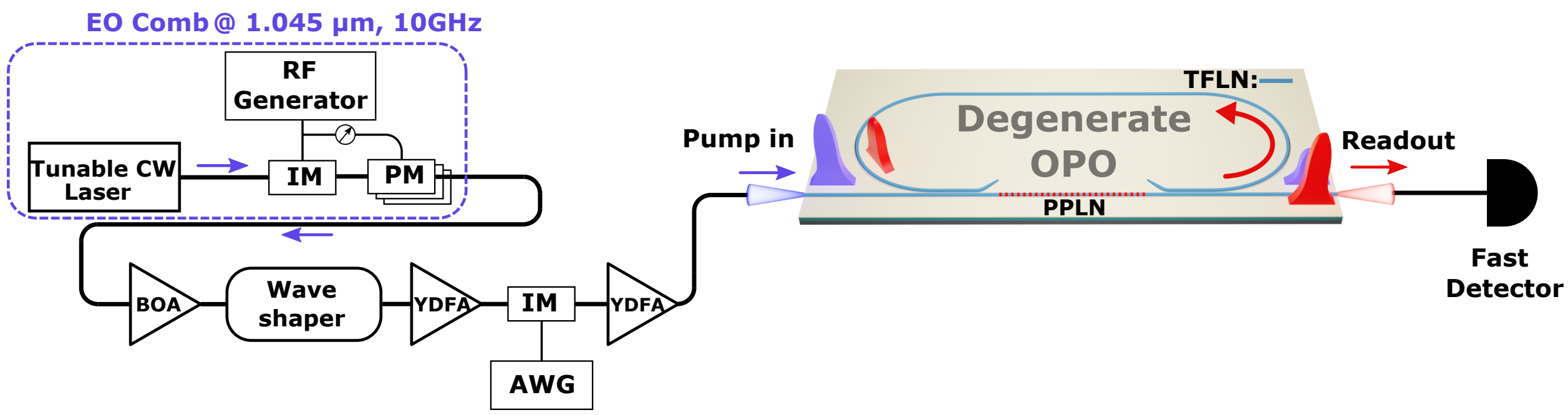

Figure S1: **Schematic diagram of the OPONN experimental setup.**

3 - Latency

The latency associated with the on-chip OPO cavity can be calculated using the following equation: Optical Latency = 1/(comb pulse repetition rate) $\times N_{\text{in}} + \tau_{\text{RT}}$, where the input comb pulse repetition rate in all cases considered in our study is $\approx$ 10 GHz. In addition, N_{in} denotes the number of nodes within the input weight vector $\mathbf{W}_{\text{in}}$. According to the above equation, the latency linearly increases with N_{in}, since the input pulses need to be repeated N_{in} times for the OPO network to operate. Finally, τ_{RT} corresponds to the roundtrip time of the OPO cavity, which is equal to $\approx$ 100 ps. We therefore conclude that the optical latency of the OPO is equal to $\tau_{\text{L1}} \approx 400$ ps for the time-series prediction and nonlinear channel equalization tasks and $\tau_{\text{L2}} \approx 600$ ps for the noisy waveform classification tasks.

We should emphasize that the end-to-end latency in our current implementation of the OPO-based neural network is limited by the latency associated with the electronic measurement (electrical cables, oscilloscope etc.) used to apply the linear output weights. However, as indicated by the Figure S4 “ened-to-end all-optical OPONN”, our approach can enable sub-nanosecond latencies via eliminating long cables and electronic connections.

To show this, we note that the total latency of this architecture consists of three components: 1- The latency associated with the OPO reservoir τ_{OPO}, which can be calculated above as $\tau_{OPO,1} = \tau_{\text{L1}} \approx 400$ ps for the time-series prediction and nonlinear channel equalization tasks and $\tau_{OPO,1} = \tau_{\text{L2}} \approx 600$ ps for the noisy waveform classification tasks.

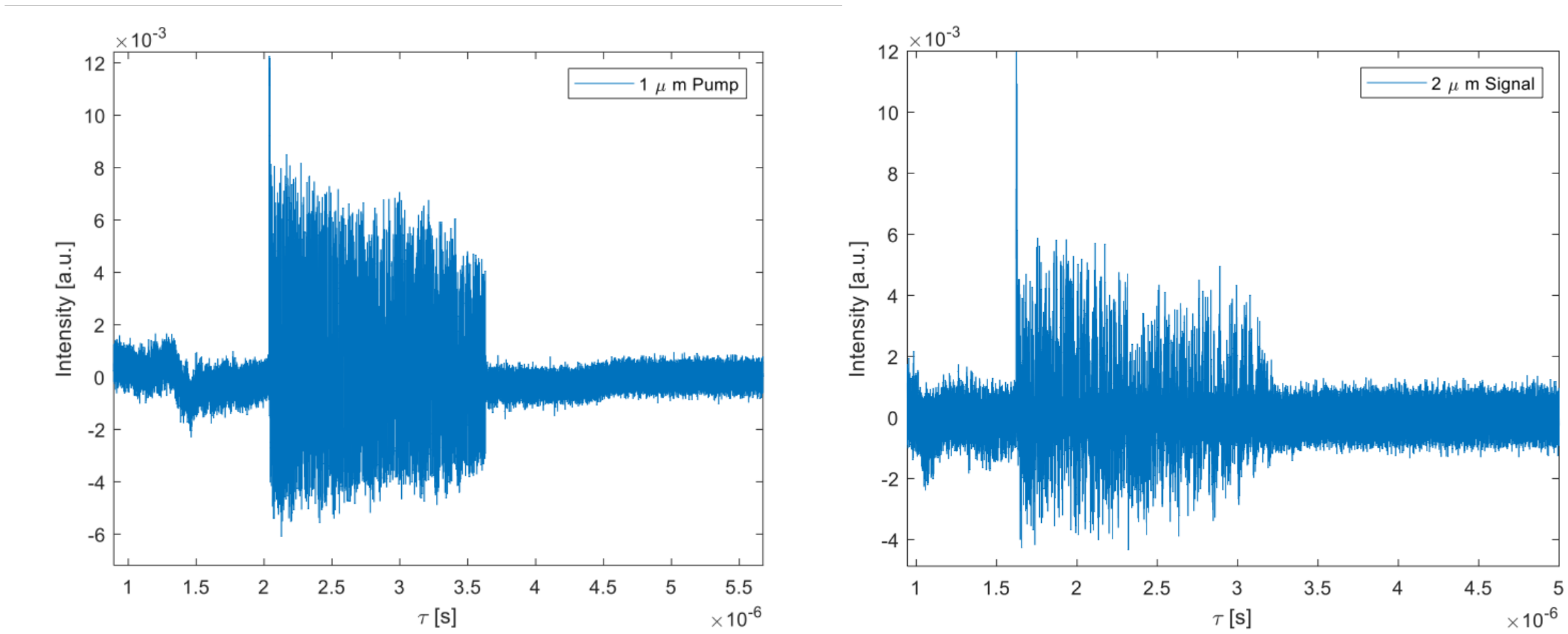

Figure S2: **Different SNR levels associated with the measured pump and signal pulses.** Example measurement traces involving the pump pulses @ 1045 nm (left) and the emitted signal pulses @ 2090 nm by the OPO (right).

2- The latency associated with the interconnection between the OPO reservoir and the all-optical output layer, $\tau_{Int} = n_g L_{Int}/c$, where L_{Int} denotes the length of the optical waveguide connecting the two structures, c is the speed of light in vacuum, and n_g is the group index of the waveguide. Given that both the OPO reservoir and the delay lines + EOMs can all be fabricated on the same TFLN chip, a typical L_{Int} can be made less than $1mm$, resulting in $\tau_{Int} < 10$ ps.

3- The latency corresponding to the linear output layer. Since this part of the architecture can also be entirely realized on TFLN in a monolithic fashion, thus allowing for compact low- V_π electro-optic modulators, the length of this section can be made less than ≈ 1 cm. This translates into $\tau_{Delay} <$ 80 ps.

Therefore, the overall latency of the proposed all-optical architecture can be calculated as: $Latency = \tau_{OPO} + \tau_{Int} + \tau_{Delay}$ < 600 + 10 + 100 = 710 ps.

4 - Energy efficiency

In our implementation, we used the output of the AWG directly (without additional amplifiers) to drive the input EOM. This RF signal delivered about 1 mW of power into the EOM. Indeed, the AWG consumes more power than this in order to generate this arbitrary signal. However, we should emphasize that in a realistic scenario the modulated light comes from an external source, and the

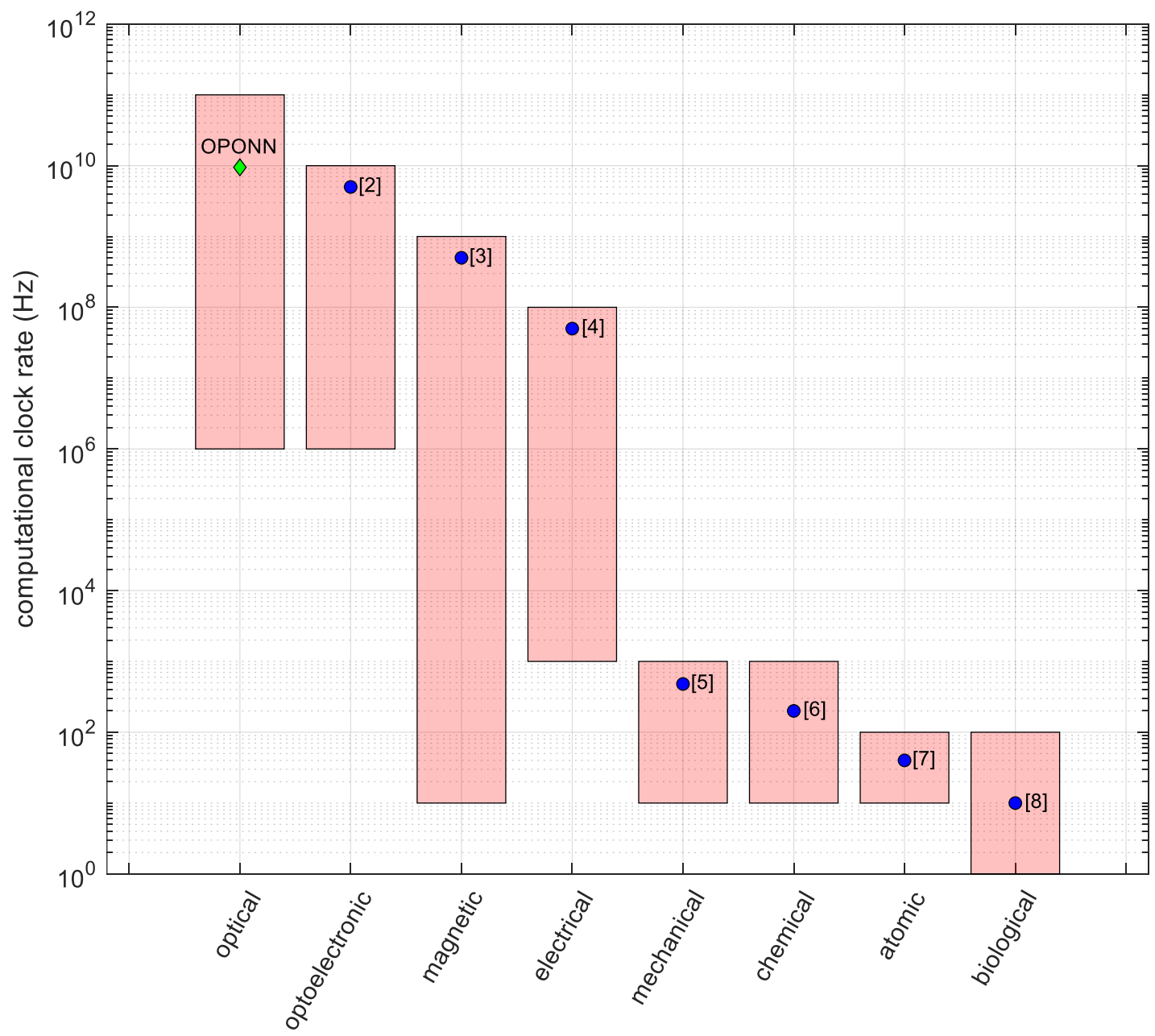

Figure S3: **Comparison of computational clock rates.** Bars show the rough order-of-magnitude ranges for clock rates in experimentally demonstrated physical recurrent networks based on different types of physical mechanisms. The marker point in each bar shows a representative state-of-the-art example for that category, numbers in brackets correspond to the reference numbers in Table 1.

electronic circuitry required for generating the data is not a part of the optical computing system. This is the case for instance in optical communications or optical signals generated by light-based sensors. Another significant source of power consumption in our implementation is the optical power associated with the pump which is roughly 10-50 mW. Finally, the digital weight processing in the current implementation is another contributor to the power consumption. The end-to-end all-optical scheme envisioned in Fig. S4 is aimed at eliminating this digital weight processing.

Overall, the energy efficiency argument of our scheme can be illustrated by comparing the two architectures shown in the schematic shown in Fig. S5. The structure shown in the left panel Fig. S5 (a) is used in many optoelectronic computing architectures (*13, 17, 53*) including reservoir computing (*54*). In such implementations, the linear operations are performed using optical interference (for instance using MZIs) while the nonlinear activation function necessary for machine learning is applied using digital electronics. As indicated in the figure, this approach requires successive

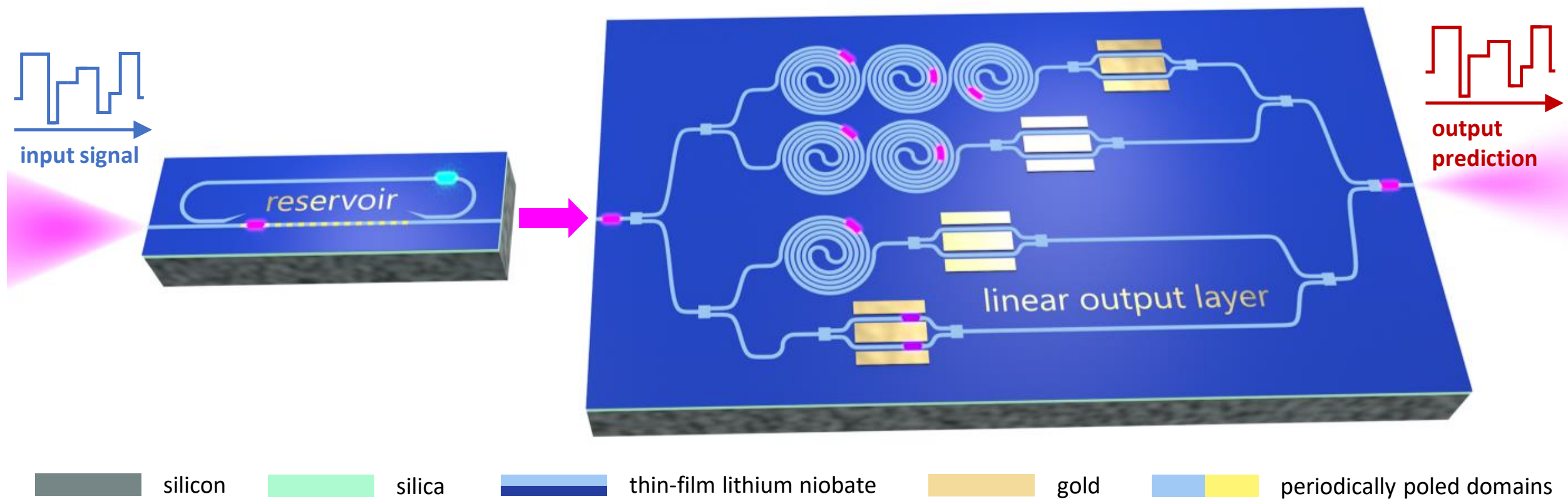

Figure S4: **An end-to-end all-optical OPONN.** Both the recurrent and linear output layers can be implemented directly in the same thin-film lithium niobate integrated platform.

optical to electrical to optical (O-E-O) conversions to implement a multilayer or recurrent architecture. Such O-E-O conversions in turn necessitate analog-to-digital and digital-to-analog converters (ADC/DAC). The operating speed of ADCs and DACs are known to be limited by their associated clock rates, which can in principle reach 10 GS/s. However, it is well known that ADC power consumption tends to grow exponentially with the effective number of bits (ENOB) and grows at least linearly with the sampling rate (*55*). For instance, a state-of-the-art ADC with 12-bit precision and 10 GS/s sampling rate can consume > 2 W of power (*56*). In comparison, the architecture employed in our study which is schematically shown in Fig. S5 panel (b), does not require successive usage of ADC/DAC since it does not involve successive O-E-O conversions. Hence, this can lead to a reduction in the energy consumption compared to conventional electro-optic architectures similar to panel (a) of this figure.

5 - Nonlinear channel equalization task

The schematic below paints a more detailed picture of the implementation of our OPO network implemented in our experiment. Regarding the nonlinear channel equalization task, we first prepared 3000 symbols of PAM4 signals that are randomly drawn from four discrete levels $S(n) \in \{-3, -1, 1, 3\}$ where $n = 1, 2, \ldots, 3000$. To emulate the effect of propagating through a dispersive, noisy and nonlinear communication channel, we modified the transmitted signal accord-

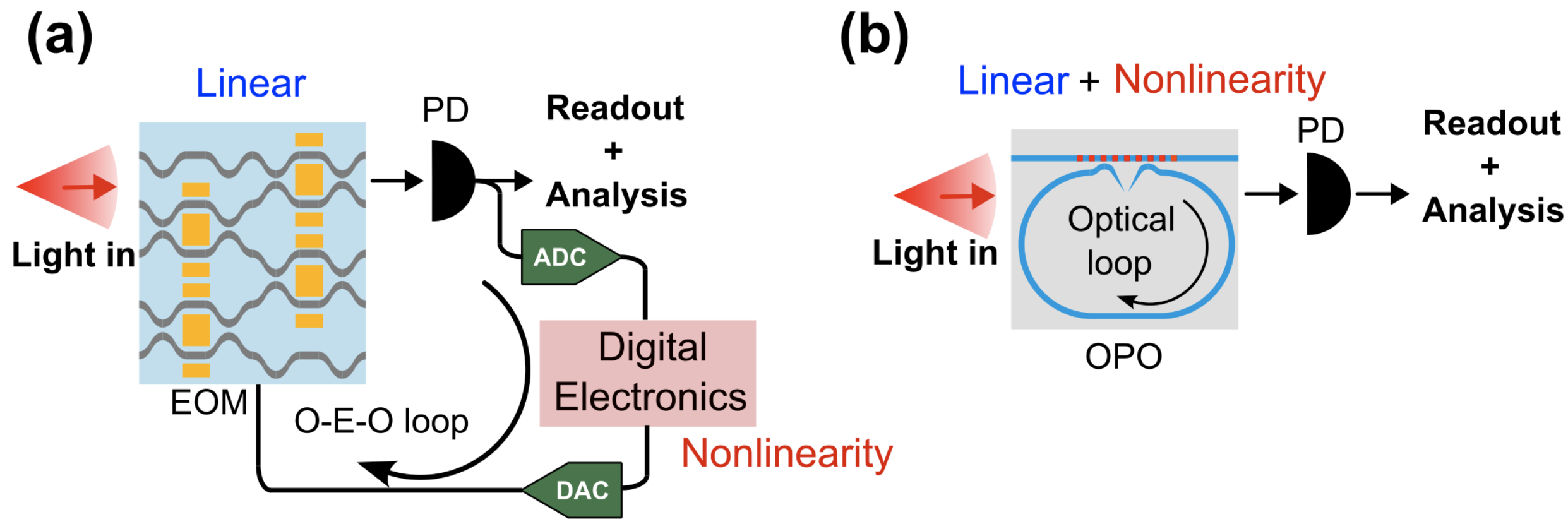

Figure S5: **Comparing hybrid vs. all optical neural network architectures.** (a) Hybrid implementation of linear and nonlinear functions. (b) Our OPONN can facilitate all-optical linear and nonlinear operations.

ing to the equation $u(n) = Q(n) + 0.036Q^2(n) - 0.011Q^3(n) + R(n)$. Here, $Q(n) = \sum_{i=-2}^{7} \alpha_i S(n-i)$ accounts for inter-symbol interference (ISI), with the values $\alpha_{-2} = 0.08, \alpha_{-1} = -0.12, \alpha_0 = 1, \alpha_1 = 0.18, \alpha_2 = -0.1, \alpha_3 = 0.091, \alpha_4 = -0.05, \alpha_5 = 0.04, \alpha_6 = 0.03, \alpha_7 = 0.01$, while $R(n)$ models additive Gaussian noise in the channel. Given this, we utilized the following cases in the receiver end and compared their performances:

Case 1: Direct detection. Using direct detection performed on the modulated pump $u(n)$, a symbol error rate (SER) of $19.3 \pm 1.6\%$ was obtained experimentally.

Case 2: Nonlinear equalization scheme via OPONN. Using the OPONN implemented as shown in the figure below, where nonlinear activation function and coherent addition was provided by the integrated OPO. In this case, using an input weight vector $\mathbf{W}_{\text{in}}$ with $N_{\text{in}} = 3$ elements and an output weight vector $\mathbf{W}_{\text{out}}$ with $N_{\text{out}} = 21$ elements (as illustrated in Fig. S6), we generate an output sequence of $o(n)$ to estimate the input signal. Using this method, we obtained an SER of $7 \pm 2.3\%$.

Case 3: Linear equalization scheme. To examine the role of the OPO cavity in the channel equalization, we also tried a linear equalization scheme. In this case, all the parameters was kept the same as Case 2, except for removing the OPO chip between the $u(n)$ and $o(n)$ signals, such that in Fig. S6 $y(n) = \mathbf{W}_{\text{in}} u(n)$. In particular, we kept the same input weight vector $\mathbf{W}_{\text{in}}$ with $N_{\text{in}} = 3$ elements and an output weight vector $\mathbf{W}_{\text{out}}$ with $\mathbf{W}_{\text{out}} = 21$ elements. In this case, we obtained an

SER of 11 ± 1.8%.

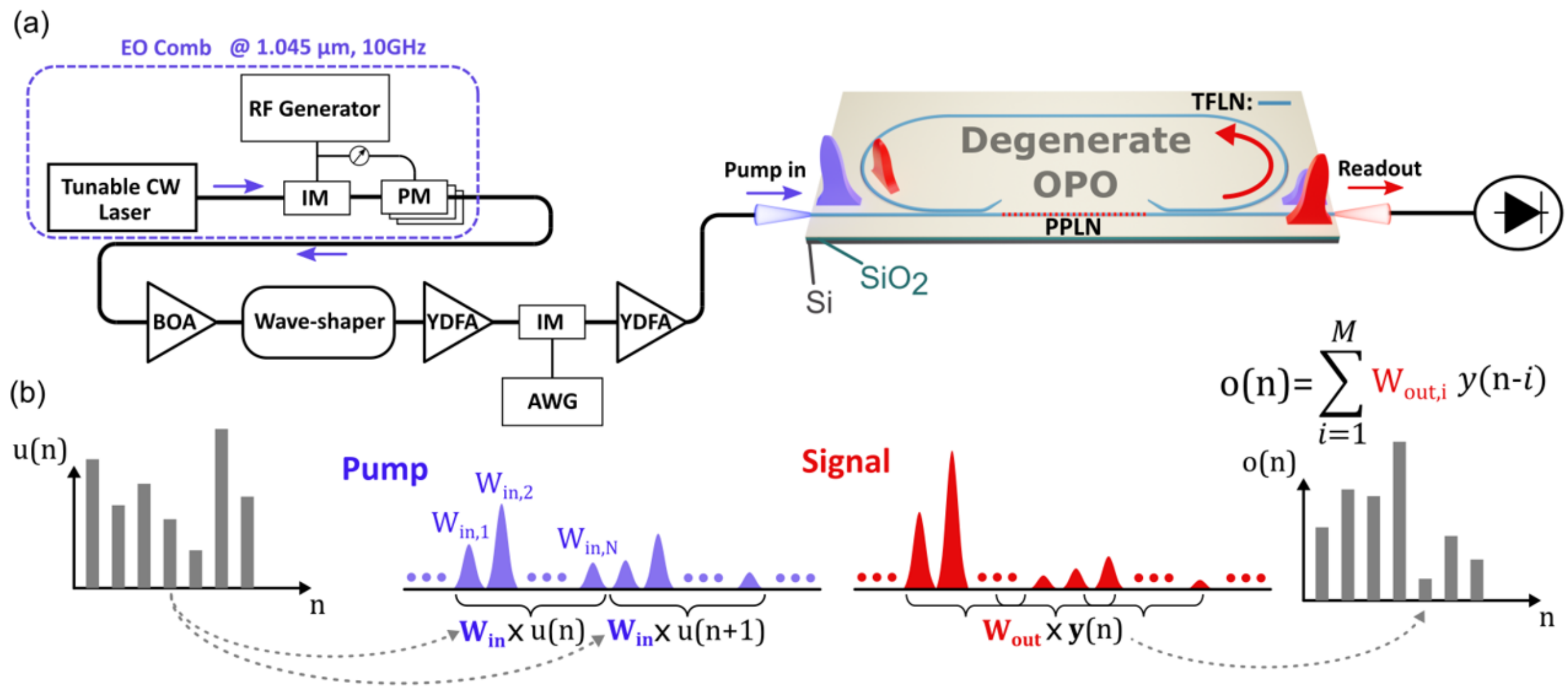

Figure S6: **Input and output weights in the OPONN.**

6 - Phase mismatch and imperfection effects

In the presence of device fabrication variations, we emphasize that our computing scheme utilizes the output of the physical OPO network to find the optimum output weights in a manner similar to physics-aware training. Therefore, if we were to change the physical OPO device for the same machine learning task, our scheme can adapt and perform successfully provided that we re-train the weights for the new device. Furthermore, for each device, the pump wavelength has to also be tuned in order to ensure degenerate conditions for the OPO.

In order to assess the performance of the OPONN in the presence of phase mismatch and **without re-training** (fixed output weights), we ran Monte Carlo simulations. Here, we assume a stochastic scenario resulting in a phase mismatch Δk corresponding to a Gaussian distribution of zero mean and a standard deviation of $2\pi \times 10^{-6}$ rad/μm. Figure S7 shows the likelihood of the normalized mean squared error (NMSE) associated with the Lorenz63 time series prediction task for this random scenario, assuming no re-training is performed to compensate for the fluctuations in the phase matching conditions.

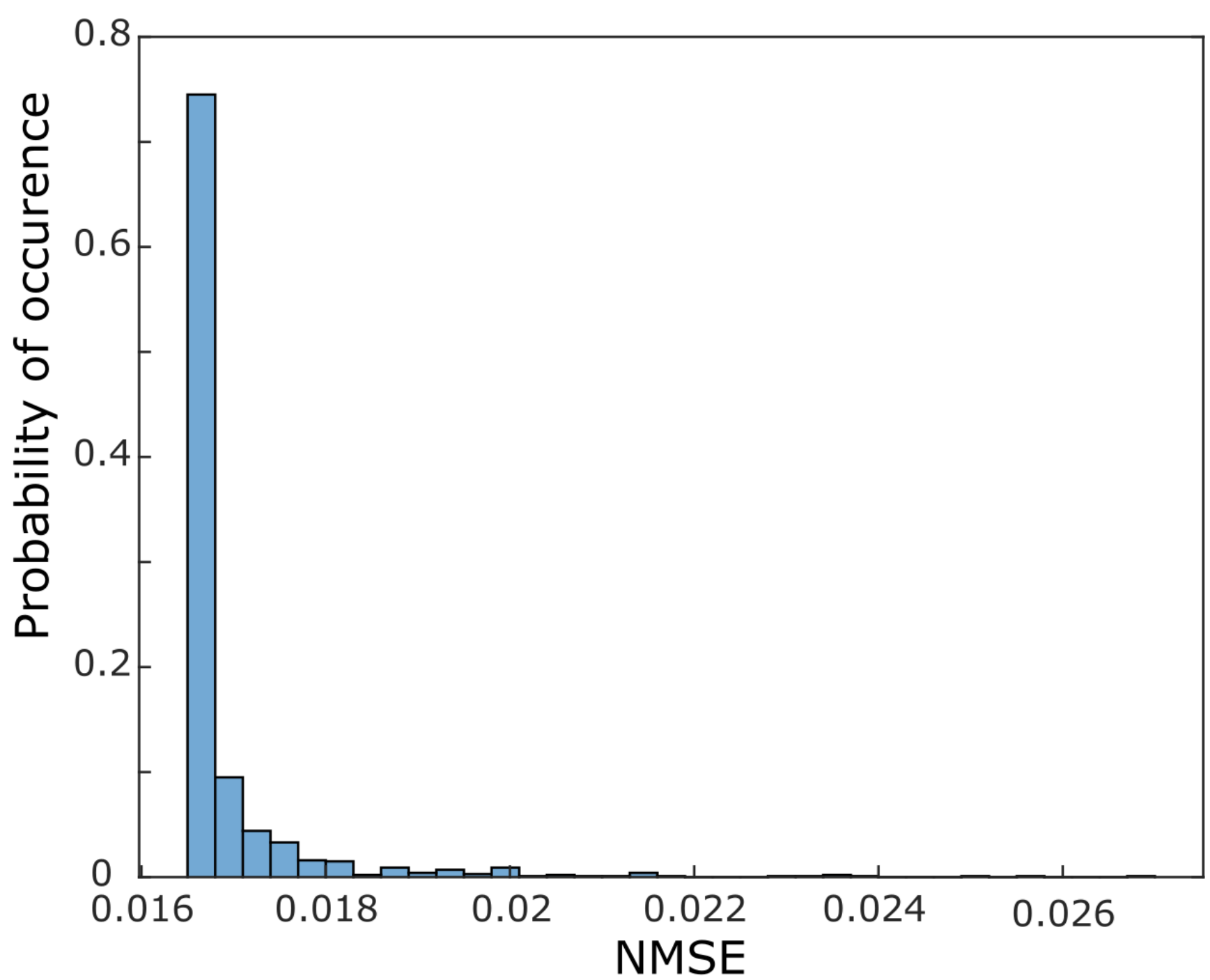

Figure S7: **Monte Carlo simulation of normalized error distribution associated with random phase mismatch in the Lorenz63 time series prediction using OPONN.**

7 - Nonlinear response of the OPO

We modeled the OPO's nonlinear response by applying a linear ramp modulation on the pump power and calculated the output signal power. The results are shown in Fig. S9 for various input/output coupling efficiencies η. As indicated by these results, the nonlinear response (in the form of a sigmoid-like function) as well as the conversion efficiency of the OPO network can indeed be modified by changing the pump power and the escape efficiency of the OPO cavity.

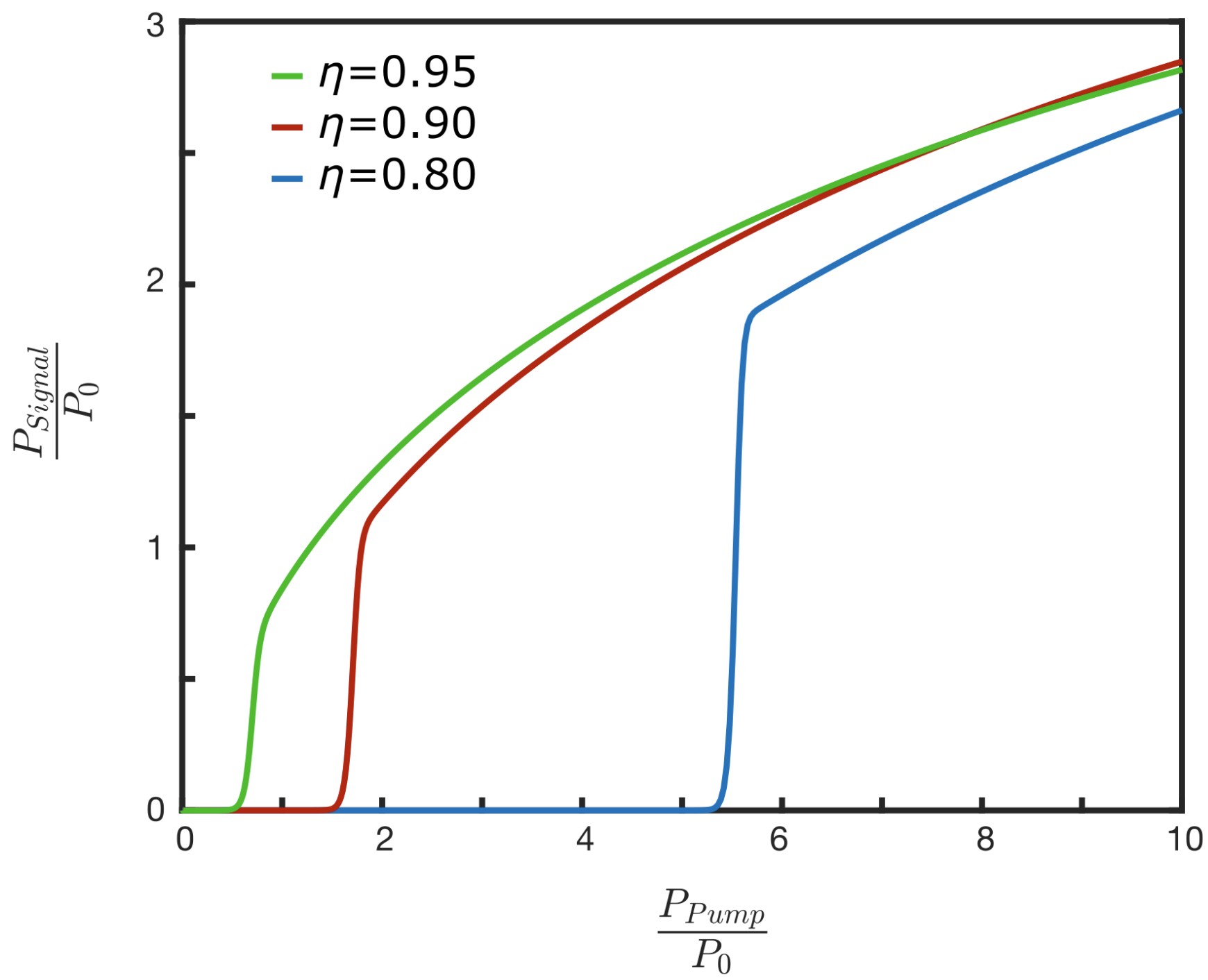

Figure S8: **Simulated nonlinear response of the OPO cavity.**

The input and output powers are normalized to an arbitrary power P_0.

The relationship between the input and the output of our OPO reservoir computer is governed by the following equations: $[p(N(n-1)+1) \ldots p(nN)] = u(n)[W_{in,1} \ldots W_{in,N}]$, where $p(i)$ is the i-th sample of the input pump to the OPO, $u(i)$ is the i-th sample of the input data to the network, and $[W_{in,1} \ldots W_{in,N}]$ denote the input weights of the reservoir that are chosen randomly. In response to this, the OPO will generate the output signal sequence $y(n) = f[p(n)]$, where $f[\cdot]$ signifies the nonlinear input/output relation associated with the OPO. Finally, the output of the OPO reservoir computing system is represented by $o(n) = \sum_{i=1}^{M} W_{out,i}\, y(n-i)$, where $[W_{out,1} \ldots W_{out,M}]$ is the static output weight vector trained using linear regression.

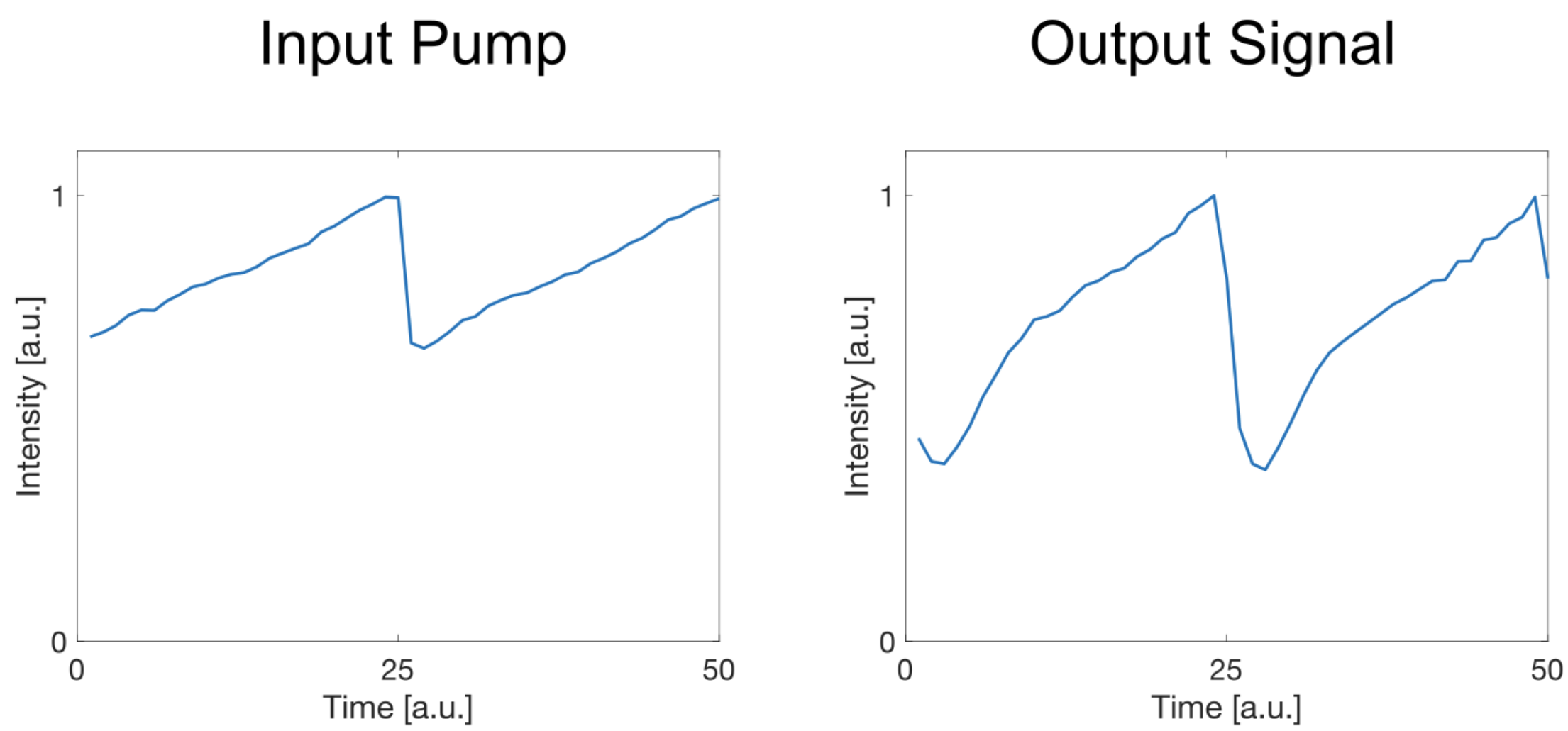

Figure S9: **Experimentally measured output response of the OPO to a linear ramp pump.**

8 - Scalability and parallel processing

We envision several schemes to go beyond a single node OPONN (*39*). Below we list a few possible solutions based on different implementations and discuss potential benefits and challenges associated with each one.

Wavelength-division-multiplexing (WDM): One possibility is to multiplex several input times series each centered around a different pump wavelength channel, similar to Ref. (*20*). Each channel will experience its corresponding feedback and nonlinear response within the OPONN cavity. The output of the OPONN can be configured using a WDM filter to separate various processing channels based on its associated signal wavelength. Given the broad gain bandwidth of our TFLN-based OPOs (as discussed in Ref. (*41*) of the manuscript), this technique can be used to incorporate many parallel channels to be processed simultaneously in an OPO-based neural network. Challenges associated with method can include coupling between various input wavelength channels that can be difficult to account for.

Time-division-multiplexing (TDM): A different approach can involve combining multiple input (pulsed) signals by assigning different time slots to each input channel, similar to Ref. (*38*). Such a scheme can be implemented using relatively simple hardware at the input and can also support a large number of processing channels (limited by the ratio between the pulsed pump repetition rate and the duration of the pulses). At the output, however, pulse pickers may be needed

to separate various TDM channels.

Coupled OPOs: Another possibility is to deploy multiple OPO cavities that are coupled to each other via for instance a common waveguide. Each OPO can be driven by a dedicated pump input. The output signals of the OPOs can be combined coherently or incoherently to yield the computation results. Compared to other schemes, this approach can provide high computational capacities by accessing large dimensional representation of the data but also poses hardware challenges associated with fabrication of multiple OPOs with matched properties including free spectral ranges (FSR) and phase matching conditions.

Ref.	Type	Platform	Tasks	Clock Rate
This Work	Optical	Integrated optical parametric oscillator	Chaotic time series forecasting, nonlinear error correction, waveform classification	9.47 GHz
(*43*)	Optoelectronic	Semiconductor fiber laser	Spoken digit recognition, chaotic time-series forecasting	5 GHz
(*44*)	Magnetic	Spin-torque nano-oscillator	Spoken digit recognition, waveform classification	500 MHz
(*45*)	Electrical	Ferroelectric field-effect transistor	Boolean logic, nonlinear time series forecasting	50 MHz
(*46*)	Mechanical	Miura-ori origami	Nonlinear filter emulation, Volterra series, nonlinear limit cycles	480 Hz
(*47*)	Chemical	Organic electro-chemical transistor	Heartbeat classification	200 Hz
(*48*)	Atomic	Boron dopants in silicon	Boolean logic, MNIST image classification	40 Hz
(*49*)	Biological	Dissociated rat E18 cortical neurons	Spike timing, random music classification	10 Hz

Table S1: Comparison of state-of-the-art experimentally reported physical recurrent networks based on different types of physical mechanisms.

Ref.	Hardware Architecture	Task	Reported Accuracy/Error	Data Rate
This Work	Integrated $\chi^{(2)}$ OPO network	Lorenz63 time series prediction<br>Mackey-Glass time series prediction<br>Nonlinear channel equalization<br>Noisy waveform classification	NMSE = 0.07<br>NMSE = 0.06<br>SER = 0.07<br>100% accuracy	9.47 GHz
(*43*)	Semiconductor laser with delayed optical feedback (time-multiplexed)	Spoken digit classification<br>Santa-Fe time series prediction	0.014% Error<br>NMSE=10.6%	$\approx$ 1 Gbyte/s
(*50*)	Optoelectronic loop (fiber-optic delay line + electronics)	NARMA10<br>Nonlinear channel equalization<br>Spoken digit classification	NMSE = 0.168<br>10^{-4} SER<br>WER=0.4%	12.5 kHz
(*33*)	Fiber loop reservoir	NARMA10<br>Nonlinear channel equalization<br>Spoken digit classification	NMSE = 0.107<br>$\approx 10^{-4} - 0.08$ SER<br>WER=0	1 MHz
(*9*)	Optoelectronic loop (fiber-optic delay line + electronics)	Spoken digit classification<br>Santa-Fe time series prediction	WER=0.04%<br>NMSE=0.124	$\approx$ 20 MHz

(*51*)	Fiber loop reservoir	Speech recognition	WER=0.04%	≈ 17.6 GHz
(*52*)	Optoelectronic loop (fiber-optic delay line + electronics)	Nonlinear channel equalization	$\approx 10^{-4} - 0.08$ SER	≈ 128 MHz

Table S2: Comparison between this work and state-of-the-art optical reservoir computers.
